# Burnout and associated factors in psychiatry residents: a systematic review

**DOI:** 10.5116/ijme.5d21.b621

**Published:** 2019-07-30

**Authors:** Min Kai Chan, Qian Hui Chew, Kang Sim

**Affiliations:** 1 Tan Tock Seng Hospital, Singapore; 2 Research Division, Institute of Mental Health, Singapore; 3 West Region, Institute of Mental Health, Singapore

**Keywords:** Burnout, psychiatry, residents, stress, support

## Abstract

**Objectives:**

This study aimed to systematically review extant data on the prevalence of burnout amongst psychiatry residents, examine the contributory factors, and consider potential ways to manage burnout.

**Methods:**

A systematic literature review was conducted on all relevant articles within Pubmed/OVID Medline and ScienceDirect digital databases from January 2000 till March 2019 that investigated burnout in psychiatry residents. Variables of interest included questionnaires used to assess burnout, the prevalence of burnout, and its clinical correlates. Articles were included if they were observational or experimental studies and involved a sample consisting solely of or a subsample of psychiatry residents. The data are summarised and presented as a narrative synthesis.

**Results:**

Twenty-two studies were included. The overall prevalence of burnout among psychiatry residents was 33.7%, which was associated with certain demographic (non-parental status), training (juniors years of training, lower priority of psychiatry as career choice, lack of clinical supervision, discontinuation from training), work (high workload, long hours, insufficient rest), and learner factors (more stressors, greater anxiety, and depressive symptoms, low self-efficacy, decreased empathic capacity, poor coping, self- medication, and use of mental health services).

**Conclusions:**

These findings suggest that interventions such as refining candidate selection, enforcement of work hour limits, enhancement of support and supervision, and equipping of stress coping skills may ameliorate burnout related to training, work, and learner factors respectively. These findings and suggestions may apply to other residency programs. However, future studies should examine burnout longitudinally and evaluate the effectiveness of different interventions in reducing burnout within psychiatry residents.

## Introduction

Recent reports including systematic reviews have observed relatively high rates of burnout amongst medical students, residents in training, and physicians ranging from 7 to 80%,^1^^-^^5^ although actual rates may vary according to discipline. The wide range of burnout rates reported is thought to be related to the instruments used, threshold criteria for burnout employed as well as specific contextual factors unique to each group across different studies.^3^ Burnout is commonly understood as a syndrome comprising of a triad of emotional exhaustion, depersonalisation and decreased personal accomplishment. It is qualitatively different from depression in that it is related to and occurs within the context of one’s work environment. Germane to this, Erschens and colleagues^3^ found that up to 75% of medical students suffered from professional burnout in their review of 12 studies. The rates amongst physicians are similarly high, with up to 60% of those in medical and surgical specialities experiencing burnout.^1^^,^^4^ Amongst residents in training, recent studies have found that up to 80% of medical and surgical residents showed evidence of burnout.^2^^,^^5^ Thus far, there has been no systematic review of burnout prevalence specifically for psychiatry residents.

Left unattended, burnout can have undesirable consequences, including disruption to work, reduced productivity, decreased job satisfaction, decreased quality of patient care, disruption of personal relationships, and increased anxiety and depression.^6^The World Federation of Mental Health specifically included a section to address issues of burnout and stress in the workplace in their 2017 report for World Mental Health Day.^7^ Similarly, the Accreditation Council for Graduate Medical Education (ACGME) recently highlighted the importance of addressing physician well-being and creating a culture of resident engagement and well-being.^8^

There are three relevant models of burnout which are worthy of consideration for medical education. The first is the stress/coping model proposed by Cherniss.^9^ It emphasises the prominence of stressors which can quickly accumulate for the novice in-training and burnout can be seen as one way of adapting or coping with the source of stress. The second is a phase model of burnout and is based on the commonly used Maslach Burnout Inventory (MBI) which cumulates eight different progressive phases of burnout by halving and combining scores of the three subscales.^10^ The third model is a conflict model with an emphasis on emotional exhaustion as the cardinal change in the context of the clash between personal aspirations and organisational needs. This can lead to depersonalisation, followed by diminished personal accomplishment, and then burnout.^11^^,^^12^

Thus, in view of the negative effects of burnout on residents’ training and personal well-being, we aim to review the extant literature to determine systematically, 1) the prevalence of burnout amongst psychiatry residents, 2) its association with various factors (including demographic, individual, work, training), and 3) consider ways to tackle burnout with the observed factors and aforementioned models of burnout in mind.

## Methods

In accordance with the guidelines from the Preferred Reporting Items for Systematic Reviews and Meta-Analysis (PRISMA),^13^^,^^14^ a systematic review was conducted with two independent reviewers searching Pubmed/OVID Medline and ScienceDirect digital databases for studies on the prevalence and clinical correlates of burnout from January 2000 till March 2019. Keywords and combinations used for the literature search were “burnout” AND “psychiatry” AND “residents” OR “trainees”.

### Eligibility Criteria

Papers were selected for inclusion if they 1) were either observational or experimental studies, 2) involved a sample consisting solely of or a subsample of psychiatry residents 3) were focused on clinical burnout experienced in training, and 4) were written in English. Papers were excluded if they 1) did not include psychiatry residents in the sample, and 2) had a sample consisting of solely undergraduate medical students.

### Study Selection

Potentially relevant articles were first screened based on abstracts by the first author (MK) to observe if they met the inclusion criteria. Following which, selected articles were then reviewed by all authors as full reports and their bibliographies screened for additional references. All differing opinions regarding the inclusion of articles were resolved through discussion.

### Data Extraction and Synthesis

For each individual study, the first author (MK) extracted variables of interest, including the number and type of subjects, socio-demographic characteristics, questionnaires used especially burnout scales used, the prevalence of burnout, and clinical correlates of burnout. The data extracted for each included study were reviewed and verified by the other authors (QH and SK). Scoring of likely attrition or reporting bias for each study was modified from Cochrane Collaboration’s tool for assessing bias in trials.^15^ Attrition bias is a bias arising from incomplete outcome data, and reporting bias is a bias arising from selective reporting of outcomes.^15^ The preceding data was organised into spreadsheets and then summarized in a table to aid comparisons between studies and independent consideration by readers. The data are then presented as a narrative synthesis in the Results.

## Results

**Figure 1:**
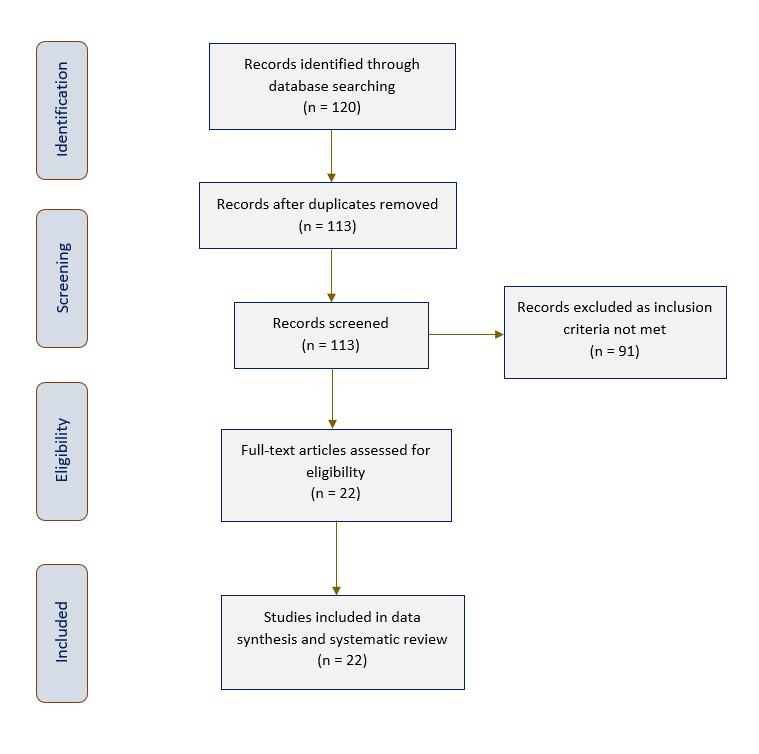
PRISMA flowchart of studies included in this review

Out of the 120 potential publications initially identified, seven duplicates were removed. Thereafter, the 113 publications were screened and 91 excluded as they did not satisfy the inclusion criteria. This resulted in a total of 22 studies being chosen for this systematic review. Figure 1 displays the PRISMA flow chart of publications selected for inclusion in this review.

The 22 included studies in this review are summarised in Appendix 1. Overall, the majority of the studies (81.8%) were conducted in the West, mostly in North America (63.6%). Females constituted 55.2% of the studies, and the overall mean age was 29.9 years old.

In terms of burnout, 13 studies (59.0% of included studies) specifically adopted a categorical definition of burnout and reported prevalence rates. The ranges of burnout prevalence related to the use of 1, 2, or 3 burnout subscale scores as cut-offs were 27.9%-87.0%, 32.0%-40.0%, 4.4%-33% respectively. Out of these 13 studies, eight studies included residents in various other medical disciplines. In studies consisting of only psychiatry residents and with clearly reported prevalence rates irrespective of threshold criteria, the overall burnout rate amongst psychiatry residents was 33.7% (883/2619).

### (A) Demographic factors

Demographic factors associated with burnout in psychiatry residents included age, gender, marital, and parental status. In terms of age, data are inconsistent in that rates of burnout were found to be associated with increased age,^16^ decreased age,^17^ or had no association with age.^18^ In terms of gender, while one study found that female residents had higher scores on the MBI subscales,^19^ two studies^20^^,^^21^ found the converse in that male residents had higher scores. However, two other studies found no association between gender and burnout.^18^^,^^22^ For marital and parental status, a study by Woodside and colleagues^21^ amongst psychiatry and family medicine residents observed that those residents with children had

lower burnout scores compared to those without children, regardless of gender. This was in agreement with the findings of Jovanović and colleagues^17^ in a relatively large international study of psychiatry residents in which severe burnout was 44% higher for psychiatry residents without children compared to those with children. However, Martini and colleagues^22^ did not find an association of burnout with marital or parental status.

### (B) Training and work-related factors

Regarding training-related factors, being a resident in junior years of training^18^^,^^20^^,^^23^ and not choosing psychiatry as a first career choice were variables associated with burnout.^17^ For example, Kealy and colleagues^18^ reported that burnout rates in PGY-4 and PGY-5 residents ranged from 16%-18% as compared to 27%-31% in PGY-2 and PGY-3 residents. Jovanović and colleagues^17^ found that within a large cohort of international psychiatry residents, the lower priority of psychiatry as one’s specialty of choice was related to burnout.  During training, burnout was also related to lack of clinical supervision,^17^ reduced satisfaction with clinical faculty,^22^^,^^23^ poorer perceived quality of supervision,^24^ reduced help-seeking from supervisors^18^ as well as discontinuation from training.^25^ In this regard, objective measures (presence/absence of clinical supervision) and subjective measures of adequate supervision (e.g. perceived quality of supervision) have been used within several studies.^17^^,^^24^ The odds of developing burnout in psychiatry residents without clinical supervision is 63% higher compared to those with clinical supervision.^17^ In addition, poorer perceived quality of supervision has been associated with an increased prevalence of burnout among residents of psychiatry and six other specialities.^24^ 

Pertaining to work-related factors, increased workload especially related to patient care responsibilities, long working hours, insufficient rest (less than 11 hours/day), and perceived wage adequacy were associated with burnout.^17^^,^^22^^, ^^24^^,^^26^^,^^27^ In one study,^17^ long working hours in residency contributed to higher levels of burnout, with the odds increasing by 9% per additional hour spent on work each week. In addition, those working more than 80 hours a week had a significantly higher prevalence of burnout (69.2%) compared to those working 80 hours or less a week (38.5%).^22^ In addition, residents on a 24-hour call versus night float system,^27^ and with a greater total number of hours spent on the electronic health record outside work^28^ were associated with higher burnout scores suggesting that striking the right balance between the work and rest is important there is evidence that reduction of work hours can reduce burnout.^22^

### (C) Learner factors

In terms of learner factors, a greater number of stressful life events, a higher level of perceived stress, fatigue, and worry were associated with higher burnout scores.^16^^,^^19^^, ^^29^^, ^^30^ Burnout was also associated with anxiety, depressive symptoms,^16^^, ^^29^ and low levels of self-efficacy.^24^ These outcomes can be linked to decreased empathic capacity and functioning,^18^^,^^31^ poor coping^18^ and lower perceived quality of patient care provided,^24^ further leading to self-prescription with psychotropic medications, and use of mental health services including psychotherapy.^16^^,^^18^ There is some evidence that a curriculum (especially resident-led) with focus on stress management, resilience building, and empathy training may be feasible and effective in reducing burnout.^32^^, ^^33^

## Discussion

There are several main findings. First, studies were mostly conducted in the West, and whilst the prevalence of burnout varies according to the threshold criteria adopted, the overall prevalence of burnout amongst studies focussing solely on psychiatry residents was 33.7%.  Second, burnout in psychiatry residents was associated with certain demographic (non-parental status), training (juniors years of training, lower priority of psychiatry as career choice, lack of clinical supervision, discontinuation from training), work (high workload, long work hours, insufficient rest), and learner factors (more stressors, greater anxiety, and depressive symptoms, low levels of self-efficacy, decreased empathic capacity, poor coping, self- medication, and use of mental health services) which can be viewed within the context of stress/coping, phase, and conflict theoretical models of burnout.

The overall prevalence rate of burnout amongst psychiatry residents was within the range of burnout rates reported amongst residents from different medical disciplines (13%-80%).^2^^,^^5^^,^^20^ The observed rates can differ depending on the instrument used as well as threshold criteria applied across the studies.^3^ Although the majority of studies included within this review adopted the Maslach Burnout Inventory, many did not report prevalence rates specifically for psychiatry residents. Various studies have also found inter-disciplinary variations of overall burnout and subscale scores, which could occur due to the differing nature of each residency program across years of training.^19^^,^^33^

### Implications of the Findings

Burnout in psychiatry residents was associated with training factors such as residency year. The stress/coping model emphasises the importance of understanding burnout as a way of coping with stressors faced during training or work, especially at an earlier stage of one’s career.^9^ Viewed within this framework, psychiatry residents in junior years of training.^18^^, ^^20^ are more vulnerable. When combined with the expectancy of heavier workload and inadequate formal supervision,^17^^, ^^24^ junior residents in training may be at an increased risk of burnout. This may be compounded by stressors outside work such as family and interpersonal issues^16^^, ^^23^ which can overwhelm the resident. There is evidence that some individuals even downplay the stressors, and may delay seeking help from supervisors.^18^^,^^30^ In addition, residents may adopt maladaptive coping measures which have been associated with burnout amongst psychiatry residents.^18^ Poor coping can result in reduced satisfaction with clinical faculty or poor perceived quality of supervision.^24^ In this early career model, possible interventions to consider include an arrangement of more customised and extended orientation for the new residents in new postings, a gradual increase of workload and training demands in earlier years of residency, and inculcation of a supportive learning environment.

Burnout in psychiatry residents was also associated with work-related factors such as long working hours. For the phase model of burnout based on MBI subscale scores,^10^ the emphasis is on the evolution of symptoms of burnout over time with different combinations of MBI subscale scores constituting progressive phases of burnout if left unattended. Within this context and related to the findings of this review, increasing workload, combined with longer work or training hours, and a more taxing 24-hours call system^17^^, ^^22^^, ^^27^ may lead to a build-up of symptoms of varying severity which can lead to full-blown burnout over time. Appropriate interventions based on this model entail organisational change related to the identification of work-related factors contributing to burnout (e.g. reducing duty hours, call system modification), ensuring adequate rest after each day of training and on-call, as well as monitoring adherence to these principles longitudinally.

Personal factors contribute to burnout in psychiatry residents as well. The conflict model serves to explain how personal, work-related, and training factors collectively contribute to the phenomenon of burnout. This model focuses on the importance of emotional exhaustion as the start of a cascade of stress responses^12^ and occurs within the context of an individual desiring to do well in the face of stressors and increased workload. The individual may cope by depersonalisation, which can further lead to decreased personal accomplishment and subsequent burnout.^12^ The findings of our review highlighted that emotional responses such as anxiety, worry, and depression may ensue in the context of overwhelming work-related, personal and interpersonal stressors.^19^^, ^^30^ This may be associated with reduced empathic capacity,^31^ decreased sense of the perceived quality of care for patients^24^ and even self-treatment with medications.^16^ Relevant measures to consider include better equipping of stress and fatigue management skills, encouragement of more adaptive coping measures, strengthening supervisor support, and empowering residents to participate in decision making to increase their sense of autonomy.

### Practical Steps for Residency Programs

What then are some practical ways to ameliorate or prevent burnout amongst psychiatry residents which are relevant for other residency programs? Whilst there may not be a single ideal way to manage burnout based on this myriad of associated factors,^35^ a 4S (selection, the standard keeping of work and learning arrangements, skills, support) framework can be considered after accounting for the findings of this review within these three models of burnout. First, the selection of appropriate candidates to join the residency is of utmost importance. There is evidence that residents who did not consider psychiatry as their first career choice are at higher risk of burnout.^17^ This may include the need for new candidates to have worked in psychiatry rotations prior to their application to join the psychiatry residency program to confirm their interest in the discipline, feedback from their previous supervisors, alongside the use of multiple mini interviews.^36^^,^^37^ Second, standard-keeping of work and learning schedules should be ensured. This includes appropriate orientation of new residents, adequate and regular clinical supervision, adherence to duty hour rules such as a limit of 80 hours per week, not working beyond stipulated working hours with adequate rest, attendances of requisite learning and supervision sessions, and tracking the compliance of training sites over time. Both areas would help to reduce the perceived stress and actual workload, especially in residents new to residency or in junior years of training.^9^ Third, skills-equipping workshops should be incorporated into the learning curriculum. Stress management techniques such as deep breathing, progressive muscle relaxation, a reminder to pace and space out the timetable of study, work, family, and leisure activities to achieve work-life balance can be reinforced. There is preliminary data to suggest that resident-led interventions, including relaxation and resilience training can be useful.^33^ This would equip them with adequate skills for self-care and empower the residents to take active steps to look after their personal well-being holistically, which encompasses the physical, psychological and social facets.^9^^,^^10^^,^^12^ Fourth, support from the people involved in the program and at work is crucial to the learner in training. This includes peers, senior residents, supervisors, and clinical faculty. A stronger support network would be helpful for all learners irrespective of seniority in training and practice.^10^^, ^^12^ 

### Limitations and Recommendations for Future Studies

There are several limitations in this review. First, participant response rates vary, and participant bias may be present as learners who are suffering from burnout may be less willing to join these studies. Second, the prevalence of burnout can vary according to the learning context, type of burnout scale used, and specific threshold criteria adopted even within the same scale. Third, future studies may want to examine other under-examined correlates of burnout such as stigma related to the discipline, personality factors, concurrent life events and aspects of the learning environment (e.g. perception of role autonomy, social support) which would enrich our understanding and suggest potential avenues of intervention to alleviate burnout in our learners. Fourth, most studies are cross-sectional in design, and longitudinal studies would be warranted to examine the changes in burnout rate and relationship with other demographic factors, training, work, and learner factors over time. Fifth, intervention studies are wanting and would allow evaluation of feasible and effective strategies to prevent or minimise burnout during the course of psychiatry residency training.

In conclusion, this review reveals that the overall prevalence of burnout in psychiatry residents is around 33.7%, and is associated with specific demographic, training, work, and personal factors. There is a dearth of longitudinal studies of burnout and studies examining the impact of interventions to prevent or reduce burnout over time. The main findings of this review, when viewed in the context of theories of burnout such as the stress/conflict, phase and conflict theories, prompted consideration of some practical ways to alleviate burnout amongst psychiatry residents which are relevant for other residency programs. Future studies are thus needed to investigate the effectiveness of different interventions within different training contexts to prevent or ameliorate the onset and impact of burnout.

## Conflict of Interest

The authors declare that they have no conflict of interest.

## Supplementary material

Supplementary file 1 Appendix 1. Summary of main findings of all included studies in this review

## References

[1] Dimou FM, Eckelbarger D, Riall TS (2016). Surgeon burnout: a systematic review.. J Am Coll Surg.

[2] Dyrbye L, Shanafelt T (2016). A narrative review on burnout experienced by medical students and residents.. Med Educ.

[3] Erschens R, Keifenheim KE, Herrmann-Werner A, Loda T, Schwille-Kiuntke J, Bugaj TJ, Nikendei C, Huhn D, Zipfel S, Junne F (2019). Professional burnout among medical students: systematic literature review and meta-analysis.. Med Teach.

[4] Sanfilippo F, Noto A, Foresta G, Santonocito C, Palumbo GJ, Arcadipane A, Maybauer DM, Maybauer MO (2017). Incidence and factors associated with burnout in anesthesiology: a systematic review.. Biomed Res Int.

[5] Lee PT, Loh J, Sng G, Tung J, Yeo KK (2018). Empathy and burnout: a study on residents from a Singapore institution.. Singapore Med J.

[6] Dewa CS, Loong D, Bonato S, Thanh NX, Jacobs P (2014). How does burnout affect physician productivity? A systematic literature review.. BMC Health Serv Res.

[7] Bahrer-Kohler S. Mental health in the workplace – burnout and stress at work. Occoquan (VA): World Federation for Mental Health (US); 2017 [cited 27 Oct 2018]; Available from: https://wfmh.global/wp-content/uploads/2017-wmhd-report-english.pdf.

[8] ACGME. Physician Well-Being: The ACGME and Beyond. Chicago (IL): Accreditation Council for Graduate Medical Education; 2018 [cited 27 Oct 2018]; Available from: https://www.acgme.org/Meetings-and-Educational-Activities/Annual-Educational-Conference/Blog/Details/ArticleID/6288/Physician-Well-Being-The-ACGME-and-Beyond.

[9] Cherniss C. Staff burnout: job stress in the human services. Vol v.2. Beverly Hills (CA): Sage Publications; 1980.

[10] Golembiewski RT, Munzenrider R, Carter D (1983). Phases of progressive burnout and their work site covariants: critical issues in od research and praxis.. The Journal of Applied Behavioral Science.

[11] Leiter MP, Maslach C (1988). The impact of interpersonal environment on burnout and organizational commitment.. J Organiz Behav.

[12] Leiter MP (1992). Burn-out as a crisis in self-efficacy: Conceptual and practical implications.. Work & Stress.

[13] Liberati A, Altman DG, Tetzlaff J, Mulrow C, Gøtzsche PC, Ioannidis JPA, Clarke M, Devereaux PJ, Kleijnen J, Moher D (2009). The PRISMA statement for reporting systematic reviews and meta-analyses of studies that evaluate health care interventions: explanation and elaboration.. PLoS Med.

[14] Moher D, Liberati A, Tetzlaff J, Altman DG (2009). Preferred reporting items for systematic reviews and meta-analyses: the PRISMA statement.. PLoS Med.

[15] Higgins JPT, Altman DG, Gotzsche PC, Juni P, Moher D, Oxman AD, Savovic J, Schulz KF, Weeks L, Sterne JAC (2011). The Cochrane collaboration's tool for assessing risk of bias in randomised trials.. BMJ.

[16] Talih F, Warakian R, Ajaltouni J, Shehab AAS, Tamim H (2016). Correlates of depression and burnout among residents in a Lebanese academic medical center: a cross-sectional study.. Acad Psychiatry.

[17] Jovanović N, Podlesek A, Volpe U, Barrett E, Ferrari S, Rojnic Kuzman M, Wuyts P, Papp S, Nawka A, Vaida A, Moscoso A, Andlauer O, Tateno M, Lydall G, Wong V, Rujevic J, Platz Clausen N, Psaras R, Delic A, Losevich MA, Flegar S, Crépin P, Shmunk E, Kuvshinov I, Loibl-Weiß E, Beezhold J (2016). Burnout syndrome among psychiatric trainees in 22 countries: Risk increased by long working hours, lack of supervision, and psychiatry not being first career choice.. Eur Psychiatry.

[18] Kealy D, Halli P, Ogrodniczuk JS, Hadjipavlou G (2016). Burnout among Canadian psychiatry residents: a national survey.. Can J Psychiatry.

[19] Goldhagen BE, Kingsolver K, Stinnett SS, Rosdahl JA (2015). Stress and burnout in residents: impact of mindfulness-based resilience training.. Adv Med Educ Pract.

[20] Prins JT, Hoekstra-Weebers JEHM, van de Wiel HBM, Gazendam-Donofrio SM, Sprangers F, Jaspers FCA, van der Heijden FMMA (2007). Burnout among dutch medical residents.. Int J Behav Med.

[21] Woodside JR, Miller MN, Floyd MR, McGowen KR, Pfortmiller DT (2008). Observations on burnout in family medicine and psychiatry residents.. Acad Psychiatry.

[22] Martini S, Arfken CL, Balon R (2006). Comparison of Burnout Among Medical Residents Before and After the Implementation of Work Hours Limits.. Acad Psychiatry.

[23] Martini S, Arfken CL, Churchill A, Balon R (2004). Burnout comparison among residents in different medical specialties.. Acad Psychiatry.

[24] Dennis NM, Swartz MS (2015). Emergency psychiatry experience, resident burnout, and future plans to treat publicly funded patients.. Psychiatr Serv.

[25] Moloney J, MacDonald J (2000). Psychiatric training in New Zealand.. Aust N Z J Psychiatry.

[26] Ferrari S, Cuoghi G, Mattei G, Carra E, Volpe U, Jovanovic N. Young and burnt? Italian contribution to the international BurnOut Syndrome Study (BOSS) among residents in psychiatry. Med Lav. 2015;106(3):172-185. 25951864

[27] Scarella TM, Nelligan J, Roberts J, Boland RJ (2017). Effect of call organization on burnout and quality of life in psychiatry residents.. Asian Journal of Psychiatry.

[28] Domaney NM, Torous J, Greenberg WE (2018). Exploring the association between electronic health record use and burnout among psychiatry residents and faculty: a pilot survey study.. Acad Psychiatry.

[29] Chaukos D, Chad-Friedman E, Mehta DH, Byerly L, Celik A, McCoy TH, Denninger JW (2017). Risk and resilience factors associated with resident burnout.. Acad Psychiatry.

[30] Benson NM, Chaukos D, Vestal H, Chad-Friedman EF, Denninger JW, Borba CPC (2018). A Qualitative analysis of stress and relaxation themes contributing to burnout in first-year psychiatry and medicine residents.. Acad Psychiatry.

[31] Park C, Lee YJ, Hong M, Jung CH, Synn Y, Kwack YS, Ryu JS, Park TW, Lee SA, Bahn GH (2016). A multicenter study investigating empathy and burnout characteristics in medical residents with various specialties.. J Korean Med Sci.

[32] Bentley PG, Kaplan SG, Mokonogho J (2018). Relational mindfulness for psychiatry residents: a pilot course in empathy development and burnout prevention.. Acad Psychiatry.

[33] Chaukos D, Chad-Friedman E, Mehta DH, Byerly L, Celik A, McCoy TH, Denninger JW (2018). SMART-R: a prospective cohort study of a resilience curriculum for residents by residents.. Acad Psychiatry.

[34] Afzal KI, Khan FM, Mulla Z, Akins R, Ledger E, Giordano FL (2010). Primary language and cultural background as factors in resident burnout in medical specialties: a study in a bilingual US city.. South Med J.

[35] Brainch N, Schule P, Laurel F, Bodic M, Jacob T (2018). Psychiatric emergency services - can duty-hour changes help residents and patients?. Psychiatr Q.

[36] Reiter HI, Eva KW, Rosenfeld J, Norman GR (2007). Multiple mini-interviews predict clerkship and licensing examination performance.. Med Educ.

[37] Alweis R, Collichio F, Milne CK, Dalal B, Williams CM, Sulistio MS, Roth TK, Muchmore EA (2017). Guidelines for a standardized fellowship letter of recommendation.. The American Journal of Medicine.

