# Supplementary material for: Burnout and associated factors in psychiatry residents: a systematic review
Source: Int J Med Educ. 2019 Jul 30;10:149–60. doi: 10.5116/ijme.5d21.b621 (PMC6766386; doi:10.5116/ijme.5d21.b621)
Supplement: Supplementary file 1 — Appendix 1. Summary of main findings of all included studies in this review [file ijme-10-149.S1.pdf]

Appendix 1. Summary of main findings of all included studies in this review

Authors (Year of Publication) Country	Aims	Participant Details	Burnout and Other Scales Used	Prevalence of Burnout	Clinical Associations with Burnout Scores	Risk of bias
Benson et al. (2018) USA	Identify stress inducing situations and stressors, stress-reducing activities that trainees utilize	- Participants: PGY1 medicine and psychiatry residents<br>- Participation rate: 60% (51/85)<br>- Gender: M = 45% (23/51)<br>- Age: 20-29 years = 76.5% (39/51), 30-39 years = 23.5% (12/51)	1) MBI<br>2) PHQ-9<br>3) Electronic stress and relaxation diaries<br>4) Demographic data	- Prevalence of burnout = 32% (16/50)	- Frequently identified themes relating to mental health issues, conflicts/disagreements in personal lives, rotation-related stressors, tendency to downplay stressors	Attrition Bias: Low<br>Reporting Bias: Low
Bentley et al. (2018) USA	To pilot an 8-week empathy training course based in relational mindfulness and assess the impact on burnout and empathy	- Participants: PGY1 psychiatry residents<br>- Participation rate: Nil<br>- Gender: M = 71.4% (5/7)<br>- Age: Nil	1) Helpful Responses Questionnaire (a measure of empathy)<br>2) MBI-HSS<br>3) Learning Experiences Questionnaire developed by authors	- Prevalence of burnout not provided	Improvement in burnout subscale and empathy scores following the course	Attrition Bias: Low<br>Reporting Bias: Low
Brainch et al. (2018) USA	Assess the impact of a recent scheduling change and decrease in overall duty hours, on resident well-being and burnout, patient wait-time and length of stay in Psychiatric Emergency Services	- Participants: Psychiatry residents<br>- Participation rate: 84.2% (32/38)<br>- Gender: Nil<br>- Age: Nil	1) MBI-GS	- EX: PGY3 > PGY 1 & PGY 4<br>- CY: PGY4 < PGY2 & PGY3	- Juniors	Attrition Bias: Low<br>Reporting Bias: Low
Domaney et al. (2018) USA	Generate preliminary data on Electronic Health Record (EHR) use and burnout among Psychiatry residents and faculty	- Participants: PGY1, 2, 3, and 4 Psychiatry residents and faculty<br>- Participation rate: 73% (40/55)<br>- Gender: Nil<br>- Age: Nil<br>-	1) MBI<br>2) Survey of risk factors:<br>• Age<br>• Gender<br>• Clinical service duties<br>• Time spent on the EHR<br>• Hours of sleep<br>• Hours of exercise	- Prevalence of burnout for:<br>• PGY1 (60%)<br>• PGY2 (87%)<br>• PGY3 (50%)<br>• PGY4 (40%)	- EE: High self-reported stress in the last month, hours spent on the EHR outside of work, total EHR time, hours reviewing EHR notes<br>- PA: Stress in the last month, total hours spent on EHR, hours writing EHR notes	Attrition Bias: Low/Moderate<br>Reporting Bias: Low
Chaukos et al. (2018) USA	Determine the feasibility of a resident-led resiliency curriculum developed by residents, for residents, and its impact on risk and resilience factors	- Participants: PGY1 medicine and psychiatry residents<br>- Participation rate: Baseline = 91% (68/75), post-intervention = 41% (31/75)<br>- Gender: Nil<br>- Age: Nil	1) MBI-HSS<br>2) PSS<br>3) PHQ-9<br>4) Functional Assessment of Chronic Illness Therapy-Fatigue Scale<br>5) PSWQ	- Prevalence of burnout at baseline: 39% (12/31)<br>- Prevalence of burnout post-intervention: 77% (24/31)	Resident-led stress management, and resiliency training curriculum is feasible with an expected increase in burnout scores in the earlier part of the internship	Attrition Bias: Low/Moderate<br>Reporting Bias: Low

			Resilience factors:<br>1) Revised Life Orientation Test<br>2) Self-Efficacy Questionnaire scale<br>3) Interpersonal Reactivity Index<br>4) Perspective-Taking subscale<br>5) The measure of Current Status – Part A<br>6) Cognitive and Affective Mindfulness Scale			
Chaukos et al. (2017)<br>USA	To investigate the hypothesized risk and resilience factors and their association with a burnout in residents	- Participants: PGY1 medicine and psychiatry residents<br>- Participation rate: 80% (68/85)<br>- Gender: M = 39.7% (27/68)<br>- Age: 25-30 years = 83.8% (57/68), 30-35 years = 16.1% (11/68)	1) MBI<br>2) PSS<br>3) PSWQ<br>4) PHQ-9<br>5) Functional Assessment of Chronic Illness Therapy<br>6) Revised Life Orientation Test (LOT-R)<br>7) Self-Efficacy Questionnaire (SEQS),<br>8) Cognitive and Affective Mindfulness Scale (CAMS-R),<br>9) Interpersonal Reactivity Index Perspective-Taking Scale (IRI-PT)<br>10) The measure of Current Status-Part A (MOCS-A)	- Overall prevalence of burnout = 27.9% (19/68)<br>- Above threshold in both DP and PA subscales = 14.7% (10/68)<br>- Above threshold in all 3 subscales = 13.2% (9/68)	- Levels of mindfulness, coping skills<br>- Levels of perceived stress, fatigue, worry, depressive symptoms.	Attrition Bias: Low/Moderate<br>Reporting Bias: Low
Scarella et al. (2017)<br>USA	To investigate the effects of changing the structure of call on residents' well-being.	- Participants: PGY2 residents who were active in the psychiatry training program<br>- Participation rate: Nil<br>- Gender: Nil<br>- Mean Age: Nil	MBI	No burnout prevalence provided	Residents on 24-h call vs. night float had increased burnout and quality of life after 1 year	Attrition Bias: Low<br>Reporting Bias: Low
Tateno et al. (2017)<br>Japan	To investigate burnout rates among psychiatric trainees	- Participants: Psychiatric residents<br>- Participation rate: 39.7% (91/227)<br>- Gender: M = 67% (61/91)<br>- Age: 31.8 +/- 4.8	1) Suicide ideation and behaviour questionnaire (SIBQ)<br>2) MBI-GS	- Prevalence of burnout = 40.0%<br>- Mean scores on MBI-GS subclass for Japanese subjects: EX = 3.0 +/- 1.5; CY = 2.3 +/- 1.4; PE = 3.7 +/- 1.1	Nil	Attrition Bias: Low<br>Reporting Bias: Low
Yrondi et al. (2017)<br>France	To conduct a multicentre observational study to determine prevalence of burnout among French anaesthesiology residents compared to French psychiatry residents	- Participants: Anaesthesiology and psychiatry residents<br>- Participation rate: Nil<br>- Gender: Anaesthesiology: M = 55%, Psychiatry: M = 33%<br>- Age: Anaesthesiology: M = 28.8 +/- 2.4, Psychiatry: M = 27.7 +/- 2.0	1) MBI	Psychiatry Residents (level of severity)<br>EE:<br>- Low: 59.5% (88/148)<br>- Moderate: 27.0% (40/148)<br>- High: 13.5% (20/148)<br><br>DP:<br>- Low: 50.0% (74/148)	Nil	Attrition Bias: Low<br>Reporting Bias: Low

				- Moderate: 31.1% (46/148)<br>- High: 18.9% (28/148)<br><br>PA:<br>- Low: 50.7% (75/148)<br>- Moderate: 26.4% (39/148)<br>High: 23.0% (34/148)		
Jovanovic et al. (2016)<br>International	To examine burnout rates among psychiatric trainees, and associated individual, educational and work-related factors	- Participants: Psychiatric trainees from 22 countries (Austria, Belarus, Belgium, Bosnia and Herzegovina, Croatia, Czech Republic, Denmark, Estonia, France, Greece, Hong Kong, Hungary, Ireland, Italy, Japan, Latvia, Portugal, Romania, Russia, Slovenia, South, Africa, and the UK<br>- Participation rate: 26% (1980/7468)<br>- Gender: Males 40.6% (803/1980)<br>- Age: 31.9 +/- 5.3	MBI	Prevalence of Burnout<br>- Overall burnout prevalence = 36.7% (726/1980),<br>- EX: 58.9% (1167/1980); CY: 45.1% (892/1980); PE: 20.2% (414/1980)	- Younger age<br>- Absence of children<br>- Psychiatry not being a first career choice<br>- Longer work hours<br>- Lack of clinical supervision<br>- Insufficient regular rest (< 11 hours daily)<br><br>No association<br>- Years of completed training	Attrition Bias: Low<br>Reporting Bias: Low
Kealy et al<br>(2016)<br>Canada	To investigate the prevalence and impact of burnout among Canadian psychiatry residents	- Participants: Psychiatry residents in Canada<br>- Participation rate: 48% (400/833)<br>- Gender: M = 69.3% (277/400)<br>- Age: ≤ 30 years = 53.1% (212/400), 31-35 years = 35.3% (141/400),<br>≥ 36 years = 11.8% (47/400)	43-item questionnaire including 1 item measuring levels of burnout symptoms (instrument highly correlated with MBI)	- Overall prevalence of burnout = 21% (84/400)	- PGY2 of training<br>- Psychotherapy usage during residency<br>- Reduced empathic functioning,<br>- Reduced seeking of help from supervisor regarding stressful patient encounters,<br>- Engagement of unhealthy coping mechanisms<br><br>No association<br>- Age<br>- Gender<br>- Location of the residency program	Attrition Bias: Low<br>Reporting Bias: Low
Park et al. (2016)<br>South Korea	Assess empathy in medical residents, including factors modifying empathy and the relationship between empathy and burnout	- Participants: Medical residents from 4 university hospitals<br>- Participation rate: 42.2% (317/751)<br>- Gender: M = 67.5% (214/317)<br>- Age: 30.44 +/- 2.98	1) Demographic data<br>2) Jefferson Scale of Empathy<br>3) MBI	- No burnout prevalence provided	- Decreased empathic capacity	Attrition Bias: Low<br>Reporting Bias: Low
Talih et al. (2016)<br>Lebanon	To investigate the prevalence of depressive symptoms, burnout, and suicidal ideation among residents	- Participants: Residents and Interns from 20 specialities including Psychiatry<br>- Participation rate: 39% (118/300)<br>- Gender: Males 52.5% (63/118)<br>- Age: NA	1) Patient Health Questionnaire (PHQ-9)<br>2) Burnout measure (BM)<br>3) Generalised anxiety disorder-7 (GAD-7) scale	- Overall burnout prevalence = 27% (32/118)	- Older age<br>- Experienced stressful personal life events over the past 12 months<br>- Depressive and anxiety symptoms<br>- Suicidal ideation	Attrition Bias: Low/Moderate<br>Reporting Bias: Low

			4) Alcohol use disorder identification test (AUDIT)<br>5) Drug abuse screening test (DAST-10)		- Self-prescribed psychotropic medications (Present/past)<br>- Current or previous use of mental health services	
Dennis et al. (2015)<br>USA	To investigate psychiatry resident burnout in emergency departments and its association with post-training publicly insured patients	- Participants: Psychiatry residents in four residency programs in North Carolina<br>- Participation rate: 51.4% (91/177)<br>- Gender: Males 40.7% (37/91)<br>- Age: 30 or above 31.9% (29/91), <30, 68.1% (62/91)	An online survey containing 2 two items abstracted from MBI to assess burnout	- No burnout prevalence provided	- Fair/poor perceived quality of provided care<br>- Lower perceived quality of supervision.<br>- Less likelihood to treat publicly insured patients after training.<br><br>Associations with Burnout subscales<br>- US Graduates associated with feelings of depersonalisation vs IMG<br>- Residents overwhelmed by patient care responsibilities (at least once per ED shift) have higher EE<br><br>No Association<br>- Year of residency<br>- Time since last emergency psychiatric rotation	Attrition Bias: Low<br>Reporting Bias: Low
Ferrari et al. (2015)<br>Italy	An international multicentre research project to estimate the burden of BS among residents in psychiatry, and identify factors contributing to its development and prevention	- Participants: Psychiatry residents from Italy<br>- Participation rate: 60% (108/180)<br>- Gender: M = 20% (21/108), 80% (86/108)<br>- Age: 30.5 +/- 3.7	- Demographic data<br>- MBI-GS<br>- Areas of Worklife Survey (AWLS)<br>- PHQ-9<br>- Suicide Ideation and Behaviour Questionnaire<br>- Big-Five Inventory-10<br>- Questionnaire on working conditions<br>- Description of activities outside work	- PE:<br>○ Moderate burnout: 36% (39/108)<br>○ Severe burnout: 20% (21/108)	- EE & C associated with:<br>○ AWLS<br>▪ Workload<br>▪ Control<br>▪ Reward<br>▪ Working group<br>▪ Fairness<br>○ PHQ-9<br>- PE associated with:<br>○ Number of papers published<br>- EE associated with:<br>Perceived wage adequacy	Attrition Bias: Low/Moderate<br>Reporting Bias: Low
Goldhagen et al. (2015)<br>USA	To investigate the hypothesis that a mindfulness-based resilience intervention would decrease stress and burnout in residents.	- Participants: Residents from 3 specialities (Family Medicine, Psychiatry, and Anaesthesia)<br>- Participation rate: 43% (47/109); Anaesthesiology = 49% (20/41); Family medicine = 75% (9/12); Psychiatry = 32% (18/56)<br>- Gender: M = 46.8% (22/47)<br>- Age: 20-29 years = 53.2% (25/47); 30-39 years = 46.8% (22/47)<br>- --2-3 one-hour sessions	60 item survey including questions from:<br>- Depression-Anxiety-Stress Scale, 21-question version (DASS-21)<br>- Oldenburg Burnout Inventory (OLBI),<br>- Mindful Attention Awareness Scale (MAAS)<br>- Cognitive Failures Questionnaire (CFQ)	- No burnout prevalence provided<br>- Impact of intervention not significant just after intervention but showed lower burnout scores at 1 month follow up	- Females<br>- Higher perceived levels of stress	Attrition Bias: Low/Moderate<br>Reporting Bias: Low

			- Perceived level of stress during residency			
Afzal et al. (2010)<br>USA	To investigate the prevalence of burnout among resident physicians and its associations	- Participants: Residents of 7 different specialities (Emergency medicine, family practice, general surgery, internal medicine, psychiatry, obstetrics/ gynaecology, and paediatrics)<br>- Participation rate: 70% (115/166)<br>- Gender: Males 58.3 % (67/115)<br>- Age: ≥ 36years = 38.3% (44/115), ≤ 35 years = 61.7% (71/115)	Questionnaire including<br>- Demographic information<br>- 22 questions from MBI	- Overall prevalence of burnout (high EE & DP, low PA scores) = 33% (38/115)<br>- High burnout subscores: High EE = 34% (39/115); High DP = 30% (34/115); Low PA = 24% (28/115)	- OB/GYN and PSY residents had high EE subscores<br>- IM residents had lower EE and PA levels as compared to other specialities<br>- OB/GYN, EM and GS residents had high DP subscores.<br>- High EE, DP and PA subscores associated with whites, primarily English-speaking and US /Canada raised residents vs. IMGs	Attrition Bias: Low<br>Reporting Bias: Low
Woodside et al. (2008)<br>USA	To investigate the relationship between burnout, work environment	- Participants: Residents in family medicine and psychiatry<br>- Participation rate: 56.0% (155/277)<br>- Gender: Males 57.5% (77/134)<br>- Age: 35 +/- 7.5	Questionnaire including:<br>- Demographic information<br>- MBI<br>- Work Environment Scale, Real Form<br>- Acculturation status	- No burnout prevalence provided	- Males<br>- Parental status protective<br>- Acculturation status<br>- Speciality (Family Medicine >Psychiatry)<br>- Younger age<br><br>No association<br>- Marital status	Attrition Bias: Low/Moderate<br>Reporting Bias: Low
Prins et al. (2007)<br>Netherlands	To investigate the prevalence of burnout among Dutch medical residents	6) Participants: Medical residents from 18 different specialties including Psychiatry<br>7) Participation rate: 54.1% (158/292)<br>8) Gender: Males 48.1% (76/158)<br>9) Age: Average age 31.9, (SD = 3.2)	Utrecht Burn-Out Scale (UBOS-C/MBI-HHS)	- Overall burnout prevalence = 13% (21/158), highest in psychiatry residents<br>- Severe burnout = 4.4% (7/158)	- Younger age<br>- Junior years of training<br>- Males<br>- Speciality: O&G had significantly higher mean scores of PA than PSY, Anaesthesiology, and IM	Attrition Bias: Low<br>Reporting Bias: Low
Martini et al. (2006)<br>USA	To investigate the implementation of work hour limits and its impact of lowering the prevalence of resident burnout	- Participants: Residents and interns in 6 specialities (General surgery, Internal medicine, Family Medicine, Obstetrics and Gynaecology, Paediatrics, Psychiatry) in all years of training<br>- Participation rate: 118/384 (31%)<br>- Gender: NA<br>- Mean Age: NA	MBI	Residents from all years of training<br>- Pre and post work hour limitations (41% →49%)<br><br>First-year residents<br>- Pre-work hour limitations are (77% →43%)	- Longer Work hours<br>- Lower satisfaction with clinical faculty<br>- Lower mood<br><br>No Association<br>- Number of nights on call<br>- Marital status<br>- Gender<br>- Presence of children<br>- Recent family stress<br>- Country of training	Attrition Bias: Low<br>Reporting Bias: Low
Martini et al. (2004)<br>USA	To investigate resident burnout in relation to work and home-related factors	- Participants: Residents and interns in 8 specialties (Dermatology, General Surgery, Internal Medicine, Family Medicine, Neurology,	MBI<br>Questionnaire:	- Overall burnout prevalence: 50%<br>Rates in:	- Higher rates among residents who were first-year residents, dissatisfied with clinical faculty, unmarried, and experiencing a family-related stress	Attrition Bias: Low<br>Reporting Bias: Low

		Obstetrics/Gynaecology, Opthalmology, and Psychiatry)<br>- Participation rate: 110/321 (35%)<br>- Gender: NA<br>- Mean age: NA		- First-year residents (77.3%) vs. the second year and above (41.8%)<br>- >80 hours per week (56.7%) vs. ≤80 hours per week (43.5%)<br>- Dissatisfied with clinical faculty (65%) vs. satisfied (40.6%)<br>- Recent family stress (58.6%) vs. no report of recent stress (38.8%)<br>- Married (40%) vs. single/divorced/other (65.2%)<br>- ≥1 child (36.4%) vs. no children (56.5%)<br>- Relocated >50 miles (71.4%) vs. did not relocate >50 miles (45.7%)	- No association between burnout and having a child or relocating	
Moloney et al (2000)<br>New Zealand	To identify factors that affect failure to complete training	- Participants: Sample A (Current trainees, All psychiatric trainees in New Zealand); Sample B (Ex-trainees, All those who had left training during the preceding 5 years without completing the training program)<br>- Participation rate: Overall 81.9% (127/155); Sample A: 87.6% (99/113); Sample B: 66.7% (28/42)<br>- Gender: Overall Males 53.5% (68/127)<br>- Age: NA	83-item Questionnaire including MBI	- No burnout prevalence provided<br>- Current psychiatric trainees scored significantly higher on each subscale of the MBI.	- Discontinuation of training	Attrition Bias: Low<br>Reporting Bias: Low

Abbreviations: CY, cynicism dimension of burnout; DP, depersonalisation dimension of burnout; EE, emotional exhaustion dimension of burnout; EX, Exhaustion; MBI = Maslach Burnout Inventory; MBI-GS, Maslach Burnout Inventory-General Survey; MBI-HSS, Maslach Burnout Inventory-Human Services Survey; PA, personal accomplishment; PE, professional efficacy; PGY, Post-graduate Year; PHQ-9, Patient Health Questionnaire 9; PSS, Perceived Stress Scale; PSWQ, Penn State Worry Questionnaire
